# (−)-Gochnatiolide B, synthesized from dehydrocostuslactone, exhibits potent anti-bladder cancer activity *in vitro* and *in vivo*

**DOI:** 10.1038/s41598-018-27036-6

**Published:** 2018-06-11

**Authors:** Yuwen Chen, Wenhua Li, Zhongqiu Zeng, Yaxiong Tang

**Affiliations:** Chengdu Institute of Biology, Chinese Academy of Sciences, Chengdu, China

## Abstract

With limited success achieved in bladder cancer patient management, novel agents are in urgent need for the purpose of therapy and prevention. As a sesquiterpenoid dimmer isolated from *Gochnatia pomculat*, (−)-gochnatiolide B has been bio-mimetically synthesized in multiple steps with a poor yield, which heavily limited the further research and clinical application. Herein, (−)-gochnatiolide B was synthesized beginning with dehydrocostuslactone in four steps with a total yield of 26%. MTT assays showed that (−)-gochnatiolide B inhibited the growth of vast majority of human cancer cells especially bladder cancer cells. Mechanistically, (−)-gochnatiolide B induced the increased expression of pro-apoptotic proteins and the decreased expression of anti-apoptosis proteins and further resulted in apoptosis of T24 cells. (−)-Gochnatiolide B induced G1 arrest which associated with SKP2 downregulation, leading to p27/Kip1 accumulation and downregulation of cyclin D1 in T24 cells. Furthermore, *in vivo* studies showed that (−)-gochnatiolide B remarkably inhibited tumor growth by 81% compared with vehicle control. Taken together, (−)-gochnatiolide B exhibits inhibitory activity against bladder cancer cells both *in vitro* and *in vivo* by inducing apoptosis, which suggests that (−)-gochnatiolide B could be an important candidate compound for prevention and treatment of bladder cancer.

## Introduction

Bladder cancer with the highest recurrence rate among solid tumors, is the most frequent malignancy of the urinary tract and the second primary cause of death in genitourinary cancer^1^. Despite current advancements in understanding of pathophysiology of the disease, treatment of bladder cancer still remains a clinically challenging problem. Therapeutic methods, including surgery, immunotherapy, chemotherapy, and radiotherapy, have limitations for invasive bladder cancer^2^. There is an urgent need to consider diverse and neoteric methods for treatment and prevention of bladder cancer.

Natural products and their derivatives have become a precious source of drug discovery in recent decades. (−)-Gochnatiolide B, a guaianolide-type sesquiterpenoid dimer, was first isolated from the root of *Gochnatia pomculat*^3^ and has previously been synthesized from a monomer dehydrozaluzanin C which was prepared from sesquiterpene natural product α-santonin in 11 steps with a low yield^4,5^. The poor content in nature and low yield of total synthesis has limited its further research and clinical application. To date, a unique class of sesquiterpene natural products has been proven to have antitumor and anti-inflammation activities. Recently, Lei and co-workers reported that ainsliadimer A, a sesquiterpene lactone dimer, targeted Cysteine 46 of IKKα/β to inhibit NF-κB signaling, leading to induction of cell death of various types of cancer cells, suppression of tumor growth *in vivo*, and induction of endotoxin-mediated inflammatory responses^6^. However, the anticancer activity and mechanism of (−)-gochnatiolide B remains unknown.

In this study, a four-steps synthetic method that began with dehydrocostuslactone was designed to synthesis (−)-gochnatiolide B with a total yield of 26%. Furthermore, exploration of biological activities showed (−)-gochnatiolide B significantly inhibited the growth of varied cancer cells including liver, lung, ovarian, colon and prostate cancer, especially bladder cancer. Mechanistically, (−)-Gochnatiolide B regulated expression of apoptosis−related proteins and induced G_1_ arrest in T24 cells. In an EJ xenograft tumor model, (−)-gochnatiolide B resulted in tumor regression by 81% compared with vehicle control.

## Results

### Synthesis of (−)-gochnatiolide B (1)

**Figure 1:**
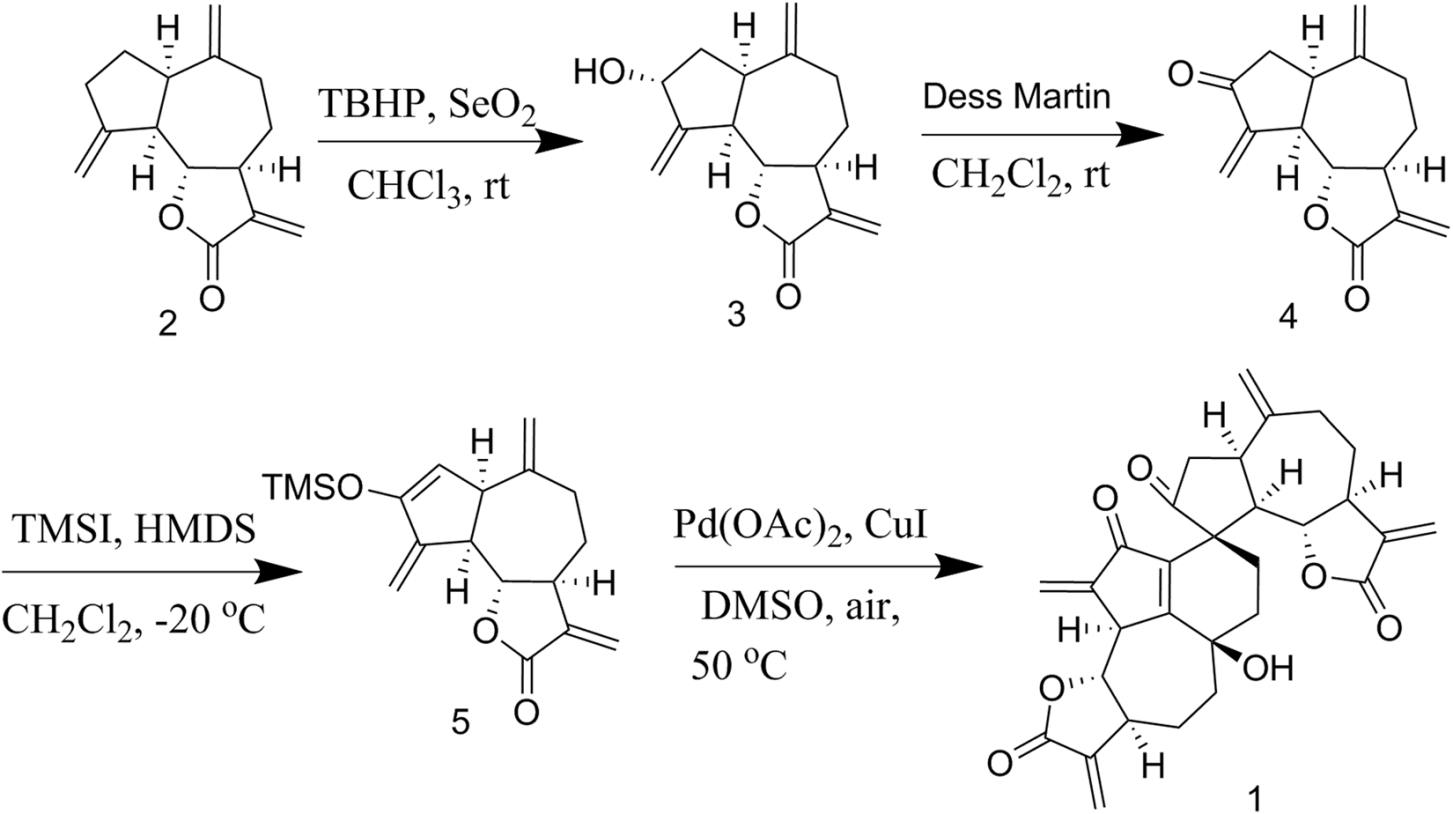
The synthetic route of (−)-gochnatiolide B. The Fig. was drawn with ChemBioDraw Ultra 12.0 by the author Y. W. C.

As shown in Fig. 1, the synthesis of (−)-gochnatiolide B (**1**) began with dehydrocostuslactone (**2**), a commercially available material of nature sesquiterpene lactones, which presents in a number of medicinal plants such as *Saussurea lappa* and *Laurus nobilis*^7–9^. Isozaluzanin C (**3**) could be prepared from (**2**) via an allylic oxidation with SeO_2_ in combination with tert-butyl hydroperoxide (TBHP, 65% in H_2_O) with yield of 73% based on recovered starting material (brsm). It is worth mentioning that several points of attack are available in dehydrocostuslactone (**2)**. For the purpose of yield improvement, the time of this reaction must be strictly controlled to avoid the further allylic oxidation of isozaluzanin C (**3)** to convert to 5α-hydroxy isozaluzanin C (Supplementary Fig. 1). Compound **3** was ulteriorly oxidized by Dess-Martin periodinane oxidation to afford dehydrozaluzanin C (**4**) in excellent yield. (−)-Gochnatiolide B (**1**) was prepared from silyl enol ether **5** which was generated by treating **4** with hexamethyldisilazane (HMDS) and trimethylsilyl iodide (TMSI), in the presence of Pd(OAc)_2_, CuCl and four times equivalent **4** through one-pot cascade transformations including inter-molecular Diels-Alder cycloaddition, Saegusa oxidation, and radical-mediated allylic oxidation^4^ with 35% yield. In general, the entire synthetic route is simple and feasible with an excellent total yield of 26%. The structure of (−)-gochnatiolide B (**1**) was confirmed by nuclear magnetic resonance spectroscopy (Supplementary Fig. 2 and Supplementary Fig. 3).

### (−)-Gochnatiolide B inhibited the growth and induced apoptosis in T24 cells

**Figure 2:**
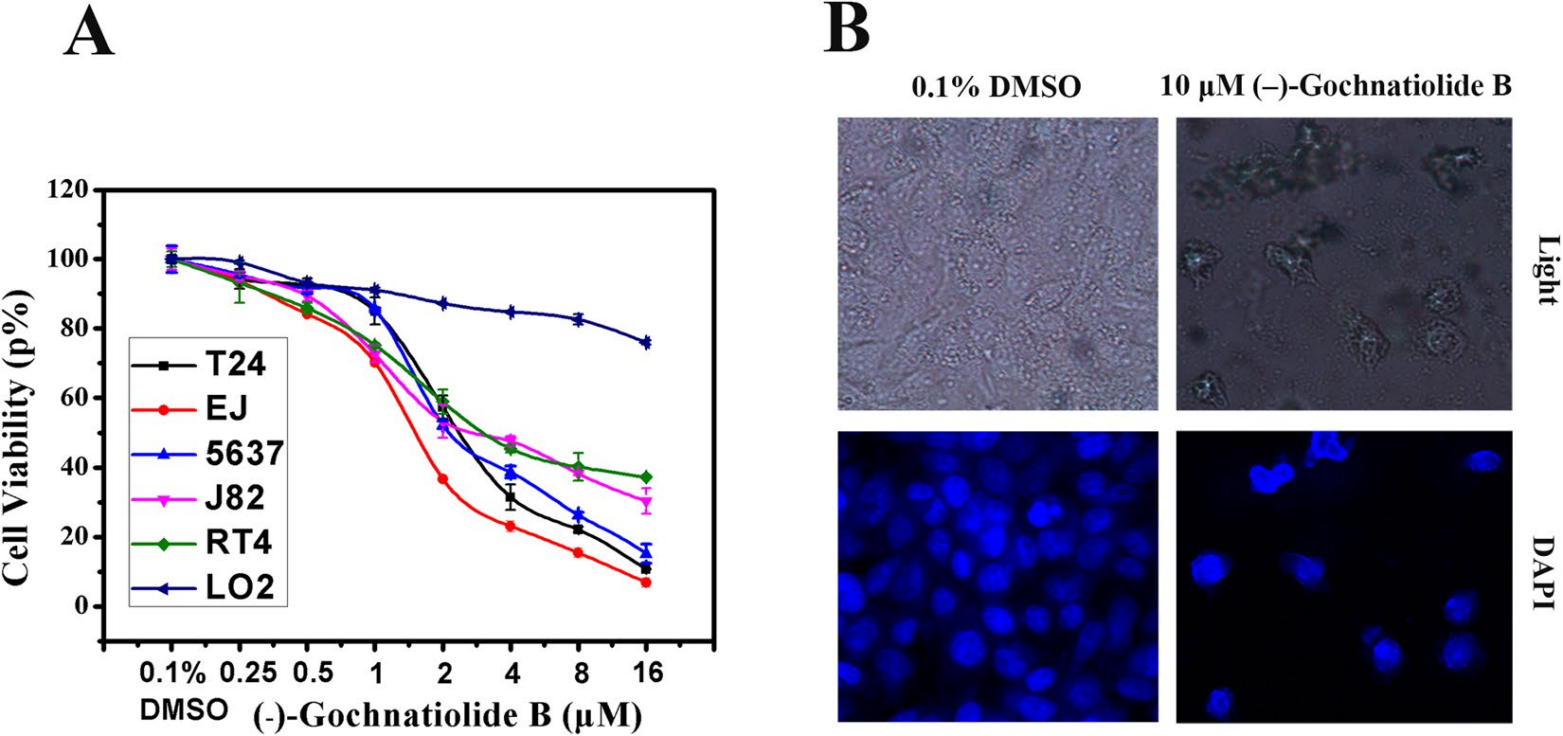
(−)-Gochnatiolide B inhibited cell growth and induced apoptosis in T24 cells. (**A**) (−)-Gochnatiolide B inhibited cell growth in bladder cancer cells including EJ, T24, 5637, J82 and RT4 cells and shown enhanced cytotoxicity than against with normal cells L02. EJ, T24, 5637, J82 and RT4 cells were treated with 0.1% DMSO or the indicated dose of (−)-gochnatiolide B for 48 h. Cell densities were measured by MTT assay. Each point is the mean ± SE of three independent experiments. Each bar represents the mean ± SE from three independent experiments. (**B**) Live cell morphology under phase contrast light microscope and DAPI staining of nuclear morphology under fluorescence microscope (magnification × 100) after T24 cells were treated with 0.1% DMSO or 10 μM (−)-gochnatiolide B for 24 h.

In order to evaluate the proliferation inhibition by (−)-gochnatiolide B, various human cancer cells were exposed to (−)-gochnatiolide B and the cell viability were measured by MTT assay. The results shown in Supplementary Fig. 4 and Supplemental Table 1, which suggest that (−)-gochnatiolide B displayed potent and broad spectrum of cytotoxicity against human cancer cell lines. As shown in Fig. 2A, (−)-Gochnatiolide B inhibited cell growth in bladder cancer cells including EJ, T24, 5637, J82 and RT4 cells and was sensitive than normal L02 cells. To investigate whether the cytotoxicity of (−)-gochnatiolide B resulted from the induction of apoptosis, T24 cells were stained with 4′,6-diamidino-2-phenylindole (DAPI) and observed under a fluorescent microscope. T24 cells treated with 10 μM (−)-gochnatiolide B exhibited significant chromatin condensation and fragmentation while these characteristics were not detected in the 0.1% DMSO-treated cells (Fig. 2B).

### (−)-Gochnatiolide B induced the apoptosis of T24 cells in the mitochondrial apoptotic pathway

**Figure 3:**
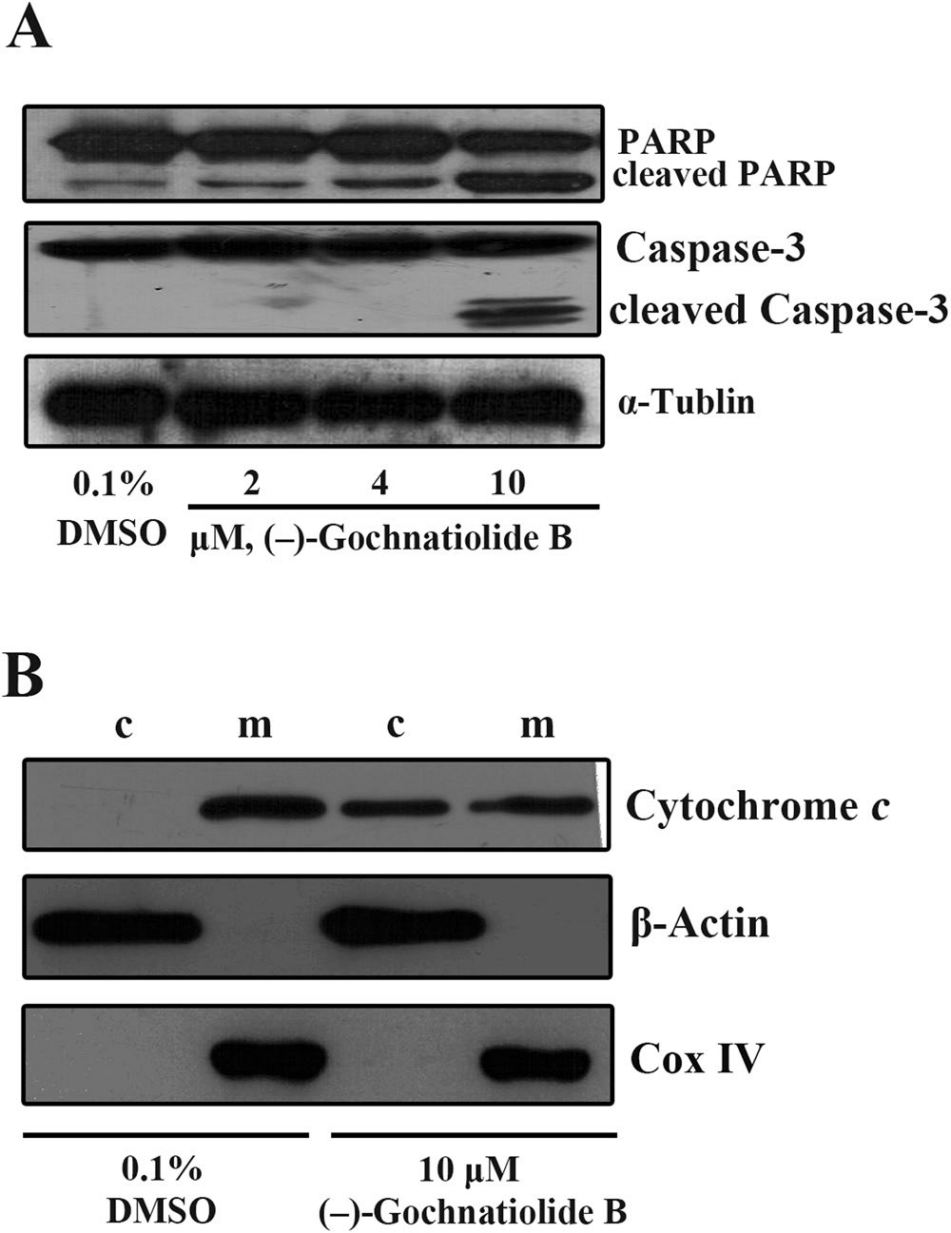
(−)-Gochnatiolide B treatment activated the cleavage of PARP and caspase-3 and resulted in cytochrome *c* release in T24 cells. (**A**) The expression of PARP and caspase-3 treated with 0.1% DMSO or (−)-gochnatiolide B (2 μM, 4 μM, or 10 μM) for 24 h in T24 cells were analyzed by Western blotting. α-Tubulin was detected as a loading control. (**B**) The expression of cytochrome *c* in mitochondria (m) and cytoplasm (c) after 0.1% DMSO or 10 μM (−)-gochnatiolide B treatment of T24 cells for 24 h were analyzed by Western blotting. β-Actin and Cox IV were used as a loading control of cytoplasm (c) and mitochondria (m) respectively.

As a sign of cell apoptosis, cleavage of PARP contributes to the further disintegration of cells following with activation of the caspase cascades. The expression of PARP and caspase-3 was analyzed by Western blotting in 0.1% DMSO or (−)-gochnatiolide B-treated T24 cells. As shown in Fig. 3A, (−)-gochnatiolide B activated caspase-3 and cleavage of PARP in a dose-dependent manner. Furthermore, to investigate whether the apoptotic effect of (−)-gochnatiolide B involved in the mitochondrial-mediated apoptotic pathway, the expression of cytochrome *c* was detected by Western blotting. As shown in Fig. 3B, (−)-gochnatiolide B induced cytochrome *c* release into the cytosol from the mitochondria. Taken together, these results suggest that the inhibitory effect of (−)-gochnatiolide B might be mediated through induction of apoptosis in mitochondrial pathways.

### (−)-Gochnatiolide B upregulated the expression of Bim and downregulated the expression of Bcl-xl, Mcl-1, XIAP and survivin

**Figure 4:**
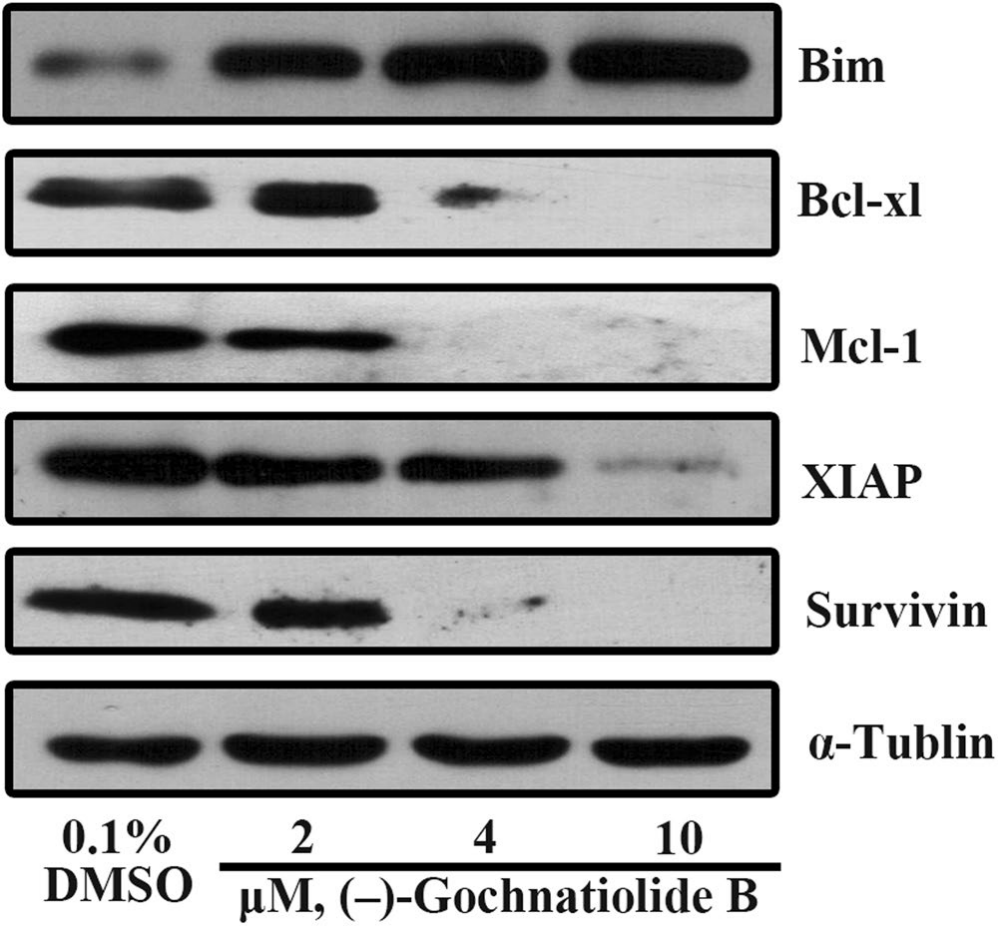
(−)-Gochnatiolide B downregulated the expression of Bcl-xl, Mcl-1, XIAP, survivin and upregulated the expression of Bim. The expression of Bim, Bcl-xl, Mcl-1, XIAP and survivin was analyzed via Western blot after T24 cells were treated with 0.1% DMSO or the indicated concentrations of (−)-gochnatiolide B for 24 h. α-Tubulin was detected as a loading control.

Members of Bcl-2 family include pro-apoptotic and anti-apoptotic proteins. A fine balance exists between these members, and the regulation of these two groups of proteins determines whether a cell survives or undergoes apoptosis^10,11^. To confirm whether the balance was lost, we examined the effect of (−)-gochnatiolide B on the expression of Bcl-2 family proteins. As shown in Fig. 4, (−)-gochnatiolide B treatment significantly increased the expression of Bim, and downregulated the expression of Bcl-xl, Mcl-1, XIAP and survivin, indicating that (−)-gochnatiolide B induced apoptosis in T24 cells through disturbing the balance between pro-apoptotic and anti-apoptotic proteins.

### Knockdown of Bim expression via ShRNA attenuated the cytotoxicity of (−)-gochnatiolide B against T24 cells

**Figure 5:**
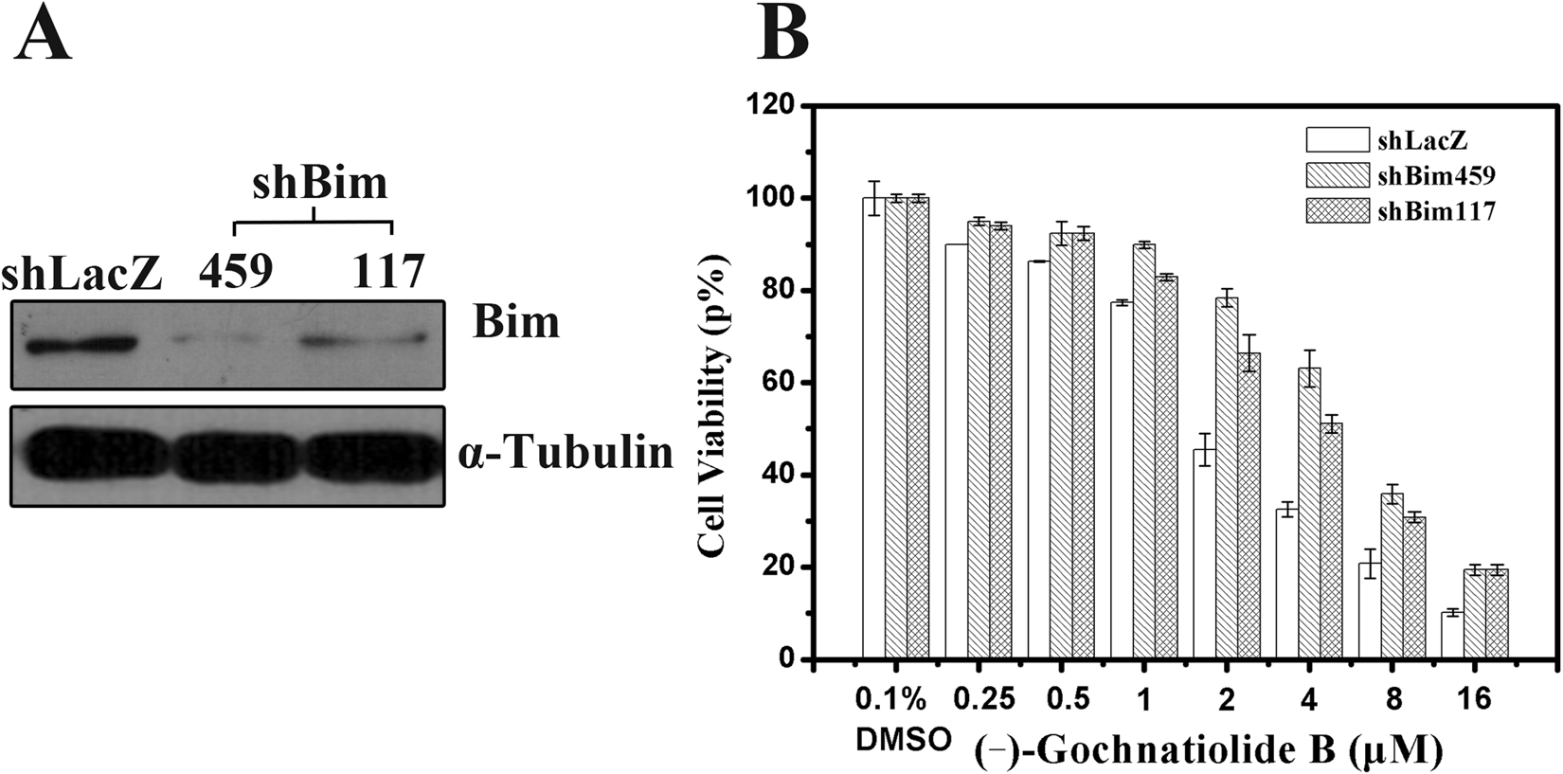
Knock-down of Bim expression via shRNA attenuated the cytotoxic effect of (−)-gochnatiolide B in T24 cells. (**A**) ShLacZ, shBim117 and shBim459 were transiently transfected into T24 cells and Bim expression was determined by Western blot. (**B**) T24 cells transfected with shLacZ, shBim459 and shBim117 were treated with 0.1% DMSO or the indicated doses of (−)-gochnatiolide B for 48 h, then cell viabilities were measured by MTT assay. Each point is the mean ± SE of three independent experiments. Each bar represents the mean ± SE from three independent experiments.

As a BH3-only protein, Bim is an immediate upstream trigger for Bax activation and initiates the BAX-mediated mitochondrial apoptosis via binding to stabilized α-helix of BCL-2 domains of BAX^12,13^. To investigate whether the expression of Bim influenced the growth-inhibitory and apoptotic effects of (−)-gochnatiolide B, Bim gene was knocked down in T24 cells via RNAi. Considering the possible off-target effects, two different Bim-targeted sequences named ShBim459 and ShBim117 were designed and the validity of the shRNA constructs and transfection efficiency was confirmed by Western blot analysis. As shown in Fig. 5A, the expression of Bim in shBim459- and shBim117-transfected T24 cells were decreased significantly. Then the shLacZ-, shBim459- and shBim117-transfected T24 cells were treated with different concentrations of (−)-gochnatiolide B as indicated respectively. The results of MTT assay demonstrated that T24 cells with knock-down expression of Bim were more resistant to the growth-inhibitory effects of (−)-gochnatiolide B (Fig. 5B). These results suggest that Bim may be a potential target for the apoptotic and growth inhibitory effects of (−)-gochnatiolide B.

### (−)-Gochnatiolide B induced G_1_ cell cycle arrests in T24 cells

**Figure 6:**
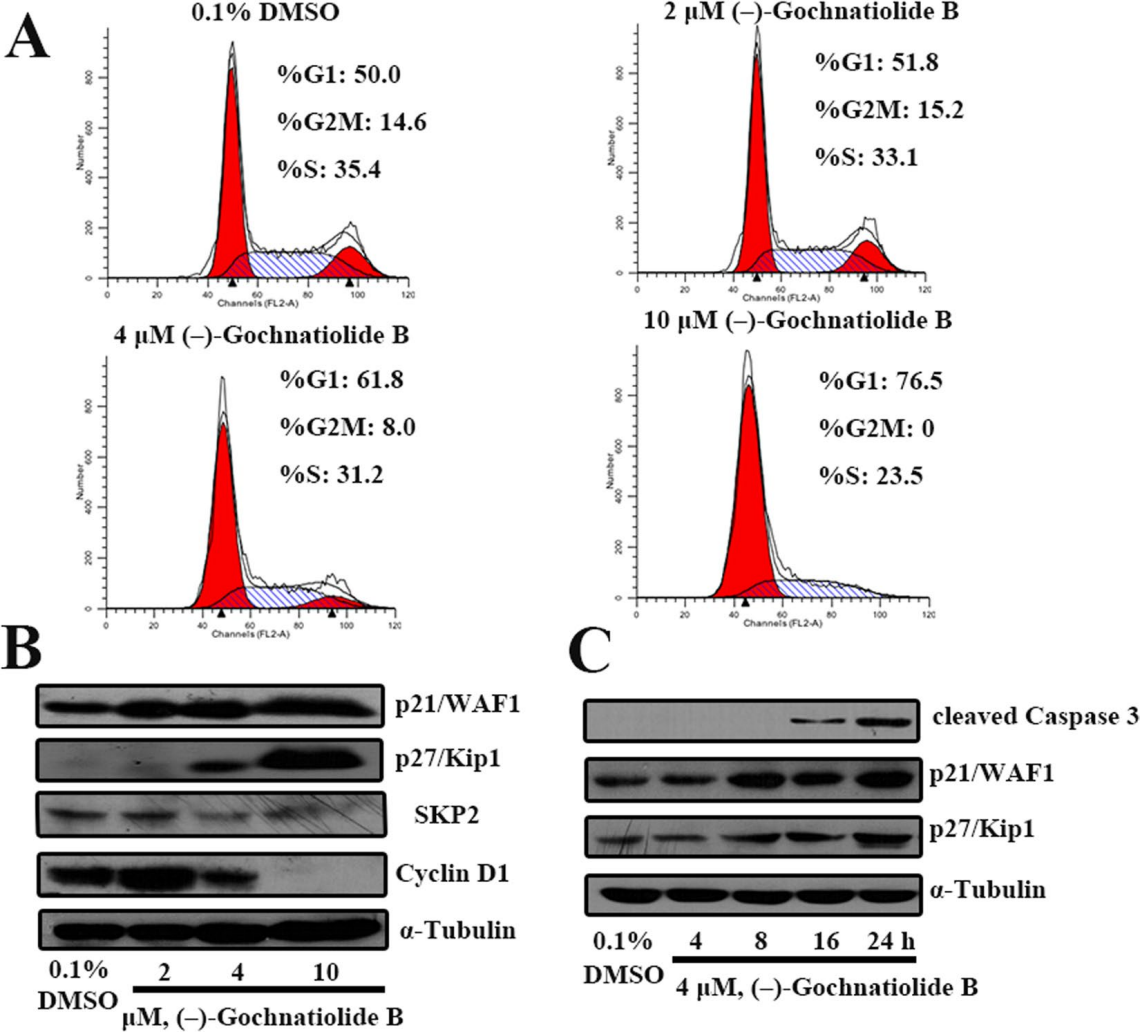
The effect of (-)-gochnatiolide B on cell cycle distribution and G1 cell cycle regulation in T24 cells. (**A**) T24 cells were treated with 0.1% DMSO, 2 μM, 4 μM or 10 μM (−)-gochnatiolide B for 24 h. Cells were collected and stained by PI, and cell cycle populations were analyzed by flow cytometry. (**B**) T24 cells were treated with 0.1% DMSO, 2 μM, 4 μM, or 10 μM (−)-gochnatiolide B for 24 h. The protein levels of p21, p27, SKP2 and cyclin D1 were analyzed by Western Blotting. α-Tubulin was detected as a loading control. (**C**) T24 cells were treated with 4 μM (−)-gochnatiolide B for different time as indicated. The protein levels of cleaved Caspase-3, p21 and p27 were analyzed by Western Blotting. α-Tubulin was detected as a loading control.

To investigate whether (−)-gochnatiolide B inhibited cell growth via the perturbation of cell cycle progression, cells were treated with different doses of (−)-gochnatiolide B for 24 h. Cell cycle distribution was analyzed through a combination of PI staining and flow cytometry. The percentage of G1 cell population increased from 50.0% in the control group to 76.5% in the 10 μM (−)-gochnatiolide B treatment group. The accumulation in G1 population with (−)-gochnatiolide B treatment at different concentrations (from 2 to 10 μM) over 24 hours was accompanied by a decrease of cells in both S and G2-M phases, indicating that (−)-gochnatiolide B induced a G1 arrest in T24 cells in a dose-dependent manner. (Fig. 6A).

As a key member during cell proliferation, cyclin D1 is synthesized rapidly and accumulates in the nucleus during the G1 phase and degrades when the cell enters the S phase^14^. Subsequently, we examined whether G1 arrest induced by (−)-gochnatiolide B treatment was associated with p21 and p27, two CDK kinase inhibitors that negatively regulate CDK2 kinase activity^15^. As expected, Western blot analysis revealed that (−)-gochnatiolide B increased the protein levels of both p21 and p27 in T24 cells (Fig. 6B). Besides, we also found that down-regulation of SKP2 is responsible for the regulation of p27 protein by (−)-gochnatiolide B treatment. (Fig. 6B). Together, these results suggest that (−)-gochnatiolide B caused G1 arrest by decreasing cyclin D1 protein expression in T24 cells, which decreased SKP2 expression level leading to accumulation of p21 and p27 proteins. To confirm whether the G1 arrest caused by (−)-gochnatiolide B followed by the induction of apoptosis, we tested the expression of cleaved caspase-3 and CDK kinase inhibitors including p21 and p27 induction after treated with 4 μM (−)-gochnatiolide B for different time in T24 cells. Western blotting analysis revealed the protein levels of both p21 and p27 were increased followed by the activation caspase-3 after (−)-gochnatiolide B treatment of T24 cells for 8 hours (Fig. 6C), which indicates that (−)-gochnatiolide B could cause G1 arrest followed by the induction of apoptosis.

### (−)-Gochnatiolide B inhibited tumor growth *in vivo*

**Figure 7:**
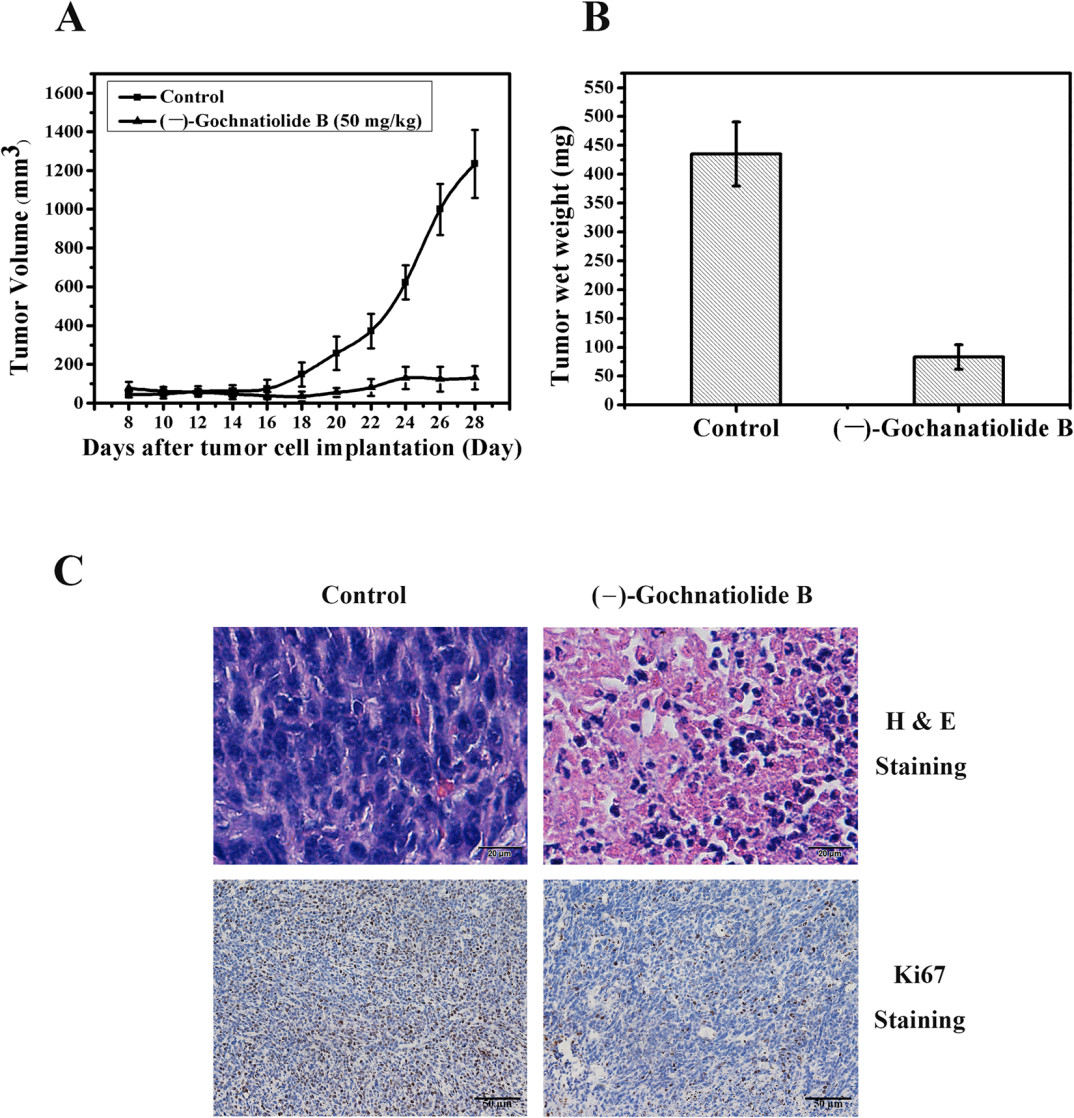
(−)-Gochnatiolide B inhibited tumor growth of EJ cells in a xenograft nude mice model. (**A**) EJ cells were concentrated to 5 × 10^6^ per 100 μl of PBS and injected into the right flank of NCR-nu/nu (nude) mice. Mice bearing EJ tumors were randomly divided, pair-matched into treatment and control groups of eight mice each, and dosing was started with vehicle or (−)-gochnatiolide B at 50 mg/kg via gavage on alternate days starting at the day of tumor growing approximately to the size of 100 mm^3^ (day 8). Tumor volumes were recorded, and presented as mean ± SE. (**B**) At the end of the study, tumors were excised from each mouse in different groups and weighed. Wet weight of tumors is represented as mean of eight tumors from individual mouse in each group. Bars, ± SE. (**C**) Hematoxylin and eosin staining in combination of Ki67 staining of tumor sections from different treatment groups including vehicle control and (−)-gochnatiolide B (50 mg/kg per every two days). Hematoxylin and eosin-stained (Scale bar = 20 μm) and Ki67-stained sections (Scale bar = 50 μm) were obtained from the tumors xenografted in mice at the end of the treatment.

To further evaluate the anti-proliferation activity of (−)-gochnatiolide B *in vivo*, we examined whether (−)-gochnatiolide B could inhibit tumor growth in a xenograft model. We constructed xenograft mouse model of EJ cells, which are similar to T24 cells, as T24 cells cannot efficiently grow in nude mice. Nude mice bearing established EJ cells were treated with vehicle or 50 mg/kg of (−)-gochnatiolide B via gavage on alternate days starting at the day of tumor growing approximately to the size of 100 mm^3^ (day 8) and ending on day 28. (−)-Gochnatiolide B treatment significantly decreased the growth rate of tumor cells compared with vehicle control (*P* < 0.05, ANOVA; Fig. 7A). The wet tumor weights in control- and (−)-gochnatiolide B-treated group recorded at the end of the treatment were 401.6 ± 124.8 mg and 83.4 ± 48.2 mg, respectively (mean ± SD; *P* < 0.05, Student’s t test; Fig. 7B). (−)-Gochnatiolide B treatment attenuated tumor growth by 81%. The body weight gain, diet, and water consumption of the (−)-gochnatiolide B-treated mice were similar to the control group of mice. In addition, the (−)-gochnatiolide B-treated mice did not show any gross abnormalities upon necropsy at the end of the treatment.

Compared with vehicle control, EJ xenograft-bearing mice treated with (−)-gochnatiolide B demonstrated remarkable changes in tissue and cell morphology as determined by histologic analysis of hematoxylin and eosin-stained sections from the tumors engrafted in mice at the end of the treatment. These changes include low cell density, as well as dark and dense nuclei, indicating mitotic catastrophe and apoptosis (Fig. 7C). To confirm whether (−)-gochnatiolide B treatment affected cell proliferation and apoptosis *in vivo*, tumor xenograft tissue sections were analyzed by immunohistochemistry of Ki67, a marker for cell proliferation. Xenograft samples from (−)-gochnatiolide B-treated mice showed a significant decrease of proliferative cells that stained positive for Ki67 when compared with controls (Fig. 7C). Together, these results suggest that the antitumor efficacy of (−)-gochnatiolide B may be achieved by inducing apoptosis.

## Discussion

Efficiently fabricating a dimeric skeleton and accurately controlling the regio- or stereo-selectivity to synthesis dimeric gochnatiolides still remain challenging problems. The total syntheses of dimeric (−)-gochnatiolide B from α-santonin have been successively accomplished in 13 steps with 0.9% overall yield by Lei and 25 steps with approximately 1% overall yield by Qin^4,16^. Compared to these methods, we synthesized (−)-gochnatiolide B from dehydrocostuslactone (2) in four steps with a total yield of 26%, which means that (−)-gochnatiolide B is obtained in a convenient, rapid and high-yield method.

Apoptosis is a main form of cell death, marked by several morphological features including condensation and fragmentation of chromatin, cell membrane blubbing and the formation of apoptotic bodies^17^. These changes are potentiated in cells via the recruitment and activation of caspases by a signaling pathway. Cleaved PARP facilitates cytolysis and undergoing apoptosis, and over-activation of PARP could deplete cellular energy. The cleavage of PARP is an important sign of apoptosis of cells and our results revealed that Caspase-3 was activated which led to PARP cleavage in response to (−)-gochnatiolide treatment B in T24 cells. Pro-apoptotic Bcl-2 family members initiate cell death through neutralizing the anti-apoptotic family members. There is evidence that Bim is essential for the execution of some apoptotic stimuli by gene targeting experiments in mice^18^. Activation of Bim by certain apoptotic stimuli such as antitumor compound treatment results in cell death, which is independent of caspase activation. Therefore, Bim activation is an upstream event in apoptosis signaling^19^. We have shown that (−)-gochnatiolide B significantly increased the expression of Bim, and subsequently induced cell death by activating the apoptosis signaling pathway.

During cell division, DNA is relatively naked and hence susceptible to damage. Cells which are actively undergoing cell cycle are targeted in cancer therapy by radiation or chemotherapeutic drugs^20^. (−)-Gochnatiolide B which disturbs the progression of cell cycle in T24 cells may be useful as a therapeutic agent of bladder cancer. In p53 mutant-type T24 cells, we have shown that (−)-gochnatiolide B induced G1 arrest by accumulation of CDK inhibitors p21 and p27 (Fig. 4B). Further studies shown that accumulation of p21 by (−)-gochnatiolide B was associated with down-regulation of SKP2 protein expression, which increased protein stability of p27 in T24 cells. Down-regulation of G1 cyclins, such as cyclin D1, by (−)-gochnatiolide B treatment in T24 cells, was also observed. Induction of cyclin D1 degradation can be a viable mechanism of action for anti-cancer drugs^21^. Independent of CDK, mounting evidence also suggests that cyclin D1 binds to nuclear receptors (including estrogen receptor α, thyroid hormone receptor, PPARγ, and AR) to regulate cell proliferation, growth, and differentiation^22–25^. (−)-Ainsliatrimer A, a guaianolide-type sesquiterpenoid similar to (−)-gochnatiolide B, which displays potent cytotoxicity against several cancer cell lines, was isolated from the aerial parts of Ainsliaea fulvioides in 2008^26^. It has been shown that (−)-ainsliatrimer A which can be biomimetically synthesized from (−)-gochnatiolide B through inter-molecular Diels-Alder cycloaddition target PPARγ^27,28^. Small molecule compounds which have similar structurse or physical and chemical properties may bind to same or similar targets^29^. Hence we speculate that the functional cellular target of (−)-gochnatiolide B might be associated with PPARγ and cyclin D1.

Compared with traditional chemotherapy and radiation therapy, targeted tumour therapy has the advantages of high specificity and low toxicity^30^. However, few targeted anti-cancer drugs that originated from natural products have been developed. In this paper, It was demonstrated that (−)-gochnatiolide B exhibited significant anti-proliferation of bladder cancer cells and inhibitory effect of tumor growth *in vitro* and *in vivo* respectively, which suggest the huge potential for drug development of (−)-gochnatiolide B and its targets. Therefore, it is great significant to identify targets of (−)-gochnatiolide B for revealing the mechanism of bladder cancer and for development of more drugs surrounded by its target. The identification and confirmatory methods such as bioinformatics, proteomics, affinity chromatography, and chip technology, chemical proteomics have become widely used for seeking small molecule protein hits by designing small molecule probes^31–33^. The main purpose is to utilize small molecule probes connected to a fluorescent tag, isotope, biotin or low affinity groups that interact with a solid phase carrier to effectively isolate the tagged biological macromolecules, which are confirmed by gel electrophoresis and mass spectrometry analysis of the target protein^31,34^. Hence, design of (−)-gochnatiolide B probes and fishing for (−)-gochnatiolide B targets based on chemical proteomics will be the core of our subsequent studies.

In summary, we have synthesized (−)-gochnatiolide B starting with a monomeric sesquiterpene lactone dehydrocostuslactone, and also demonstrated potent inhibitory activity of (−)-gochnatiolide B against proliferation of human cancer cell lines. (−)-Gochnatiolide B up-regulated Bim expression and down-regulated Mcl-1, XIAP and survivin expression, which resulted in activation of the mitochondrial-mediated apoptotic pathway to induce apoptosis of T24 cells. In p53 mutant-type T24 cells, (−)-gochnatiolide B induced accumulation of p21 and p27 proteins and decreased the expression of SKP2 and cyclin D1 followed by subsequent G1 arrest. Additionally, (−)-gochnatiolide B reduced tumor growth *in vivo*. Based on these findings, we propose that (−)-gochnatiolide B could be an effective therapeutic drug for patients who suffer from bladder cancer.

## Materials and Methods

### Instruments, reagents, and cell lines

Anhydrous Na_2_SO_4_ was used to dry organic extracts and all volatiles were removed under reduced pressure. All reaction mixtures and column eluents were monitored by thin layer chromatography (TLC) using commercial glass-backed TLC plates and the plates were visualized under UV light at 254 nm or stained with I_2_, phosphomolybdic acid, anisaldehyde or potassium permanganate. Flash chromatography was performed by 300–400 mesh silica gels. ^1^H and ^13^C NMR spectra (400 MHz) were routinely obtained on a Bruker Avance 300 machine with CDCl_3_ as the solvent. Chemical shifts were reported in parts per million relative to CDCl_3_ (1 H, δ 7.26; 13 C, δ 77.00). All other chemicals were of analytical grade.

HO8910 cells (TCHu 24) were obtained from the Shanghai Cell Bank of the Chinese Academy of Science (Shanghai, China). L02 and Mahlavu cells were obtained from Dr. J. Fan (Department of Liver Surgery, Liver Cancer Institute, Zhongshan Hospital, Fudan University). Bladder cancer EJ cells were obtained from Dr. X. L. Zi (Department of Urology and Chao Family Comprehensive Cancer Center, University of California). The other cancer cell lines were purchased from ATCC. L02, Mahlavu J82 (HTB-1) and HO8910 cells were cultured in DMEM with 10% fetal bovine serum (FBS), while HepG2 (HB-8065), H460 (HTB-177), A549 (CCL-185), SKOV3 (HTB-77), EJ, T24 (HTB-4), RT4 (HTB-2), 5637 (HTB-9), LOVO (CCL-229), HCT116 (CCL-247) and PC3 (CRL-1435) cell were cultured in RPMI 1640 supplemented with 10% FBS. Antibodies against α-tubulin, Caspase-3, PARP, cytochrome *c*, Cox IV, β-actin, Bim, Bcl-xL, XIAP, survivin, p21/WAF1, p27/Kip1, SKP2, cyclin D1 and cleaved Caspase-3 were obtained from Cell Signaling. SuperSignal West Pico Chemiluminescent detection reagents were purchased from Thermo Scientific. 3-(4,5-dimethylthiazol-2-yl)-2,5-diphenyltetrazolium bromide (MTT) and propidium iodide (PI) were purchased from Sigma.

### Procedures for the preparation of (−)-gochnatiolide B

#### Synthesis of isozaluzanin C (3)

Tert-butyl hydroperoxide (70% (v/v) in H_2_O, 9.4 mL) was slowly dropwised to a solution of dehydrocostuslactone (**2**) (2.50 g, 10.88 mmol) in CHCl_3_ (360 mL). SeO_2_ (282 mg, 2.54 mmol) was added and the mixture was stirred under the room temperature for 8 h. The reaction mixture was filtered through silica gel to stop the reaction and washed with EtOAc (100 mL), 10% NaOH aqueous solution (50 mL) and brine (50 mL × 2). The organic layer was dried over Na_2_SO_4_, filtered, and concentrated in vacuo adsorbed onto silica gel. Dry flash chromatography (EtOAc/PE = 8:1–2:1, v/v) produced the recoverd dehydrocostuslactone (**2**) (1.01 g, 40%) and isozaluzanin C (**3**) (1.15 g, 73%, brsm) as a light yellow slury. ^1^H NMR (400 MHz, CDCl_3_) δ1.36–1.40 (m, 1 H), 1.87–1.90 (m, 1 H), 2,11–2.23 (m, 2 H), 2.50–2.54 (m, 1 H), 2.83–2.90 (m, 1 H), 3.08–3.16 (m, 2 H), 3.89–3.94 (m, 1 H), 4.67–4.73 (m, 1 H), 4.80 (s, 1 H), 4.94 (s, 1 H), 5.30 (s, 1 H), 5.37 (s, 1 H), 5.52 (d, *J* = 3.5 Hz, 1 H), 6.24 (d, *J* = 3.5 Hz, 1 H).

#### Synthesis of dehydrozaluzanin C (4)

A solution of isozaluzanin C (**3**) (1.15 g, 4.67 mmol) in CH_2_Cl_2_ (35 mL) was dropwisely added to a solution of Dess-Martin periodinane (2.97 g, 7.02 mmol) in CH_2_Cl_2_ (13 mL). After 1 h, the resulting mixture was slowly added to 1.3 M NaOH (67 mL) and stirred for 15 min. The combined organic layers were extracted with CH_2_Cl_2_ (10 mL × 2), washed with water (20 mL), brine (20 mL) and dried over Na_2_SO_4_, then concentrated in vacuo. Flash chromatography (EtOAc/PE = 4:1, v/v) produced dehydrozaluzanin C (4) (1.12 g, quant.) as a white solid. ^1^H NMR (400 MHz, CDCl_3_) δ 1.40–1.52 (m, 1 H), 2.19–2.22 (m, 1 H), 2.27–2.32 (m, 1 H), 2.56–2.66 (m, 3 H), 2.98–3.07 (m, 1 H), 3.11 (td, J = 8.6, 2.2 Hz, 1 H), 3.26 (tt, J = 8.9, 3.0 Hz, 1 H), 4.01 (t, J = 9.1 Hz, 1 H), 4.60 (s, 1 H), 4.94 (s, 1 H), 5.58 (d, J = 3.1 Hz, 1 H), 5.87 (d, J = 2.7 Hz, 1 H), 6.25 (d, J = 3.1 Hz, 1 H), 6.30 (d, J = 3.5 Hz, 1 H).

#### Synthesis of (−)-gochnatiolide B (1)

HMDS (420 μL, 2.01 mmol) and TMSI (280 μL, 2.01 mmol) were slowly added in sequence to a solution of dehydrozaluzanin C (**4**) (100 mg, 0.41 mmol) in dry CH_2_Cl_2_ (10 mL) at −20 °C under dry argon. After 10 min, cooled sat.NaHCO_3_ (10 mL) was added to the reaction mixture, and the resulting mixture was stirred vigorously at room temperature for 5 min until the brown color disappeared. The resulting mixture was added with H_2_O (10 mL), extracted with CH_2_Cl_2_ (20 mL × 3) and the combined organic layers were washed with brine (20 mL × 3), dried over Na_2_SO_4_ and concentrated in vacuo to afford silyl enol ether **5** as yellow slurry which was used directly without further purification.

To silyl enol ether 5 was added dehydrozaluzanin C (**4**) (400 mg, 1.64 mmol), Pd(OAc)_2_ (92 mg, 0.41 mmol), DMSO (10 mL), and a solution of CuI (4.1 mg, 0.41 mmol) in DMSO (2 mL). The resulting mixture was sonicated and stirred at 50 °C for 20 h under air. The reaction mixture was diluted with EtOAc (100 mL), filtered through celite, and washed with EtOAc (50 mL). The filtrate was washed with H_2_O (150 mL), brine (150 mL × 2), and the aqueous layers were extracted with EtOAc (100 mL × 2). The combined organic layers were dried over Na_2_SO_4_ and concentrated in vacuo. The residue was purified by column chromatography (silica gel, EtOAc/PE = 1:3–1:1, v/v) to afford the recovered dehydrozaluzanin C (**4**) (130.6 mg, 33%) and (−)-gochnatiolide B (**1**) (72.1 mg, 35%) as a white solid. ^1^H NMR (400 MHz, CDCl_3_) δ 1.42–1.54 (m, 1 H), 1.71 (ddd, J = 13.6 Hz, 6.4 Hz, 3.6 Hz, 1 H), 1.83–1.97 (m, 1 H) 2.04–2.26 (m, 7 H), 2.29–2.35 (m, 1 H), 2.57–2.70 (m, 2 H), 2.78–2.87 (m, 1 H), 2.99–3.04 (m, 1 H), 3.22–3.28 (m, 1 H), 3.32–3.39 (m, 2 H), 3.85 (d, J = 10.9 Hz, 1 H), 4.18 (t, J = 9.1 Hz, 1 H), 4.32 (t, J = 10.2 Hz, 1 H), 4.72 (s, 1 H), 5.08 (s, 1 H), 5.53 (d, J = 2.9 Hz, 1 H), 5.57 (d, J = 2.8 Hz, 1 H), 6.13 (s, 1 H), 6.20 (m, 2 H), 6.25 (d, J = 3.1 Hz, 1 H). ^13^C NMR (100 MHz, CDCl_3_) δ 20.8, 25.7, 31.9, 35.8, 36.2, 39.4, 40.0, 43.4, 44.6, 49.5, 49.6, 51.0, 52.3, 68.2, 81.1, 83.9, 114.1, 119.2, 121.7, 122.2, 138.4, 138.8, 141.0, 142.8, 150.0, 168.9, 169.4, 169.7, 193.5, 221.7.

#### MTT assay

A density of 2 × 10^4^ cells per well were plated in 24-well culture plates. After 24 h, the medium was removed and 500 μL fresh medium containing 0.1% DMSO or different concentrations of (−)-gochnatiolide B as indicated was added to 24-well culture plates and incubated for 48 h. MTT was added at a final concentration of 1 mg/mL and incubated for 3 h. The absorbance was determined at 570 nm and the ratio of viability (%) was calculated.

#### DAPI staining

T24 cells were incubated for 24 h with 0.1% DMSO (negative control), or 10 μM (−)-gochnatiolide B. The culture medium was removed and the cells were fixed with 4% paraformaldehyde for 30 min and then stained with DAPI (4,6-diamino-2-phenyl indole) for 5 min. The cells were washed gently with PBS buffer, fixed on a glass slide, and observed and photographed under a fluorescence microscope.

#### Flow cytometry analysis of cell cycle distribution

After treatment with 0.1% DMSO (negative control), or 2 μM, 4 μM, or 10 μM (−)-gochnatiolide B for 24 h, T24 cells were fixed with 70% (v/v) alcohol overnight at −20 °C, and then the cells were washed with PBS and stained with 50 μg/mL PI for 30 min. Approximately ten thousand events were collected for flow cytometric analysis, and the distribution of G1, S, and G2-M phases of the cell cycle was determined using BD FACScan flow cytometer.

#### Western blot analysis

Total protein was obtained from T24 cells treated with 0.1% DMSO or (−)-gochnatiolide B. Denatured protein lysates (100 μg) were resolved by 8–15% SDS-PAGE and transferred to nitrocellulose (NC) membranes. The membranes were probed with indicated primary antibodies at 4 °C overnight and then with horseradish peroxidase-conjugated secondary antibody for 1–2 h. Finally, the enhanced chemiluminescence detection system was used to detect protein signals.

#### Measurement of the cytochrome *c* release from mitochondria

T24 cells were treated with 0.1% DMSO (negative control) or 10 μM (−)-gochnatiolide B for 24 h. Cytochrome *c*-releasing apoptosis assay kit was used to prepare mitochondria and cytosol. Briefly, cytosol extraction buffer was added to suspend cells and incubate on ice for 10 min. Homogenate of the cell suspension was obtained by Dounce homogenizer and centrifuged at 700 g for 10 min. The supernatant was then collected and centrifuged at 10 000 g for 30 min at 4 °C. The resulting supernatant (cytosolic fraction) and pellet (mitochondrial fraction) were used for Western blot analysis.

#### RNA interference

The Bim targeting sequence can be obtained using an RNAi designer program from Invitrogen, and the clones expressing short hairpin RNA were constructed using pENTR™/U6 entry and pBLOCK-iT™3-DEST vectors according to the BLOCK-iT™ RNAi and Gateway® Technology (Invitrogen), as previously described^35^. The RNAi target sequence for Bim (named shBim459 and shBim117) were 5′-GGAGACGAGTTTAACGCTTAC-3′ and 5′-GGTAATCCTGAAGGCAATCAC-3′ respectively, and for LacZ (named shLacZ, the negative control) it was 5′-GCTACACAAATCAGCGATTT-3′. The three target sequences were submitted to a BLAST search to ensure that only the Bim or LacZ genes were targeted. The constructs were transfected into T24 cells at approximately 70% confluency in 6-well dishes using BioT (Bioland Scientific, LLC).

#### Subcutaneous tumor model

The methods were conducted in accordance with the approved guidelines of the Good Experimental Practices adopted by the Chengdu Institute of Biology, Chinese Academy of Sciences. All experimental procedures and animal collection were conducted under the permits approved by the Committee for Animal Experiments of Chengdu Institute Academy of Sciences, China. (−)-Gochnatiolide B was formulated in 1% DMSO and 7% emulsifier EL/alcohol (3:1, v/v) in 0.9% saline and given by gavage. Purchased from Experimental Animal Center of Sichuan University, NCR-nu/nu (nude) mice were implanted 5 × 10^6^ EJ cells per 100 μL PBS and injected s.c. into the right flank of each mouse. When tumors reached approximately 100 mm^3^ in size, mice were stochastically divided into treatment and control groups of eight mice each. The vehicle or (−)-gochnatiolide B at 50 mg/kg was injected via gavage on alternate days. The tumor size, health and survival of the mice were monitored three weekly throughout the study. Tumor sizes were measured with a Vernier caliper. The tumor volume was calculated according to the formula: 0.5236 × *L*_1_ × (*L*_2_)^2^, where *L*_1_ is the long axis and *L*_2_ is the short axis of the tumor. At the end of the experiment, tumors were excised and weighed, blood was collected and all were stored at −80 °C until additional analysis.

#### Immunohistochemical staining for Ki67

The immunohistochemical staining for H & E and Ki67 performed as previously described^36^. The Ki-67 staining was conducted using the labeled streptavidin-biotin method. Tumor tissues were fixed in 10% phosphate-buffered formalin, paraffin-embedded, and sectioned. Using 10 mM sodium citrate (pH 6.0) retrieved antigen at 95 °C for 5 min. Sections were incubated with anti-Ki67 antibody in PBS for 2 h at room temperature then overnight at 4 °C. Slides were incubated with a biotinylated secondary antibody and counterstained with Harris hematoxylin then photographed using a light microscope. Negative control samples were exposed to a secondary antibody with a similar IgG isotype to the primary antibody.

#### Statistics

Student’s *t* test was used to examine the difference between treatment and control group in cell viabilities and cell cycle population experiment. One-way analysis of variance (ANOVA) was used in antitumor experiment to test the difference in tumor size. All date in this study was expressed as the mean value ± SD. *P* < 0.05 was considered to be statistically significant.

### Data availability

The authors declare that all data supporting the findings of this study are available within the article and its Supplementary Information flies, or are available from the corresponding author upon request.

## Electronic supplementary material

Supplementary Information
