# Supplementary material for: (−)-Gochnatiolide B, synthesized from dehydrocostuslactone, exhibits potent anti-bladder cancer activity in vitro and in vivo
Source: Sci Rep. 2018 Jun 11;8:8807. doi: 10.1038/s41598-018-27036-6 (PMC5995859; doi:10.1038/s41598-018-27036-6)
Supplement: Supplementary file 1 — Supplementary Information [file 41598_2018_27036_MOESM1_ESM.pdf]

(–)-Gochnatiolide B, synthesized from dehydrocostuslactone, exhibits potent anti-bladder cancer activity *in vitro* and *in vivo*

Yuwen Chen[1, 2], Wenhua Li[1, 2], Zhongqiu Zeng[1], Yaxiong Tang[1*]

[1]Chengdu Institute of Biology, Chinese Academy of Sciences, Chengdu, China.

[2]Yuwen Chen and Wenhua Li contributed equally to this work

[*]Correspondence information: Professor Yaxiong Tang, Chengdu Institute of Biology, Chinese Academy of Sciences, Chengdu, Sichuan, People's Republic of China. E-mail: tangyx@cib.ac.cn

Supplemental Fig. 1

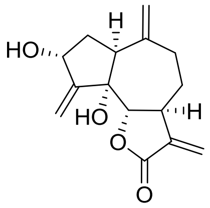

5α-hydroxy isozaluzanin C

Supplemental Fig. 1 The chemical structure of 5α-hydroxy isozaluzanin C. The Fig. was drawn with ChemBioDraw Ultra 12.0 by the author Y. W. C.

Supplemental Fig. 2

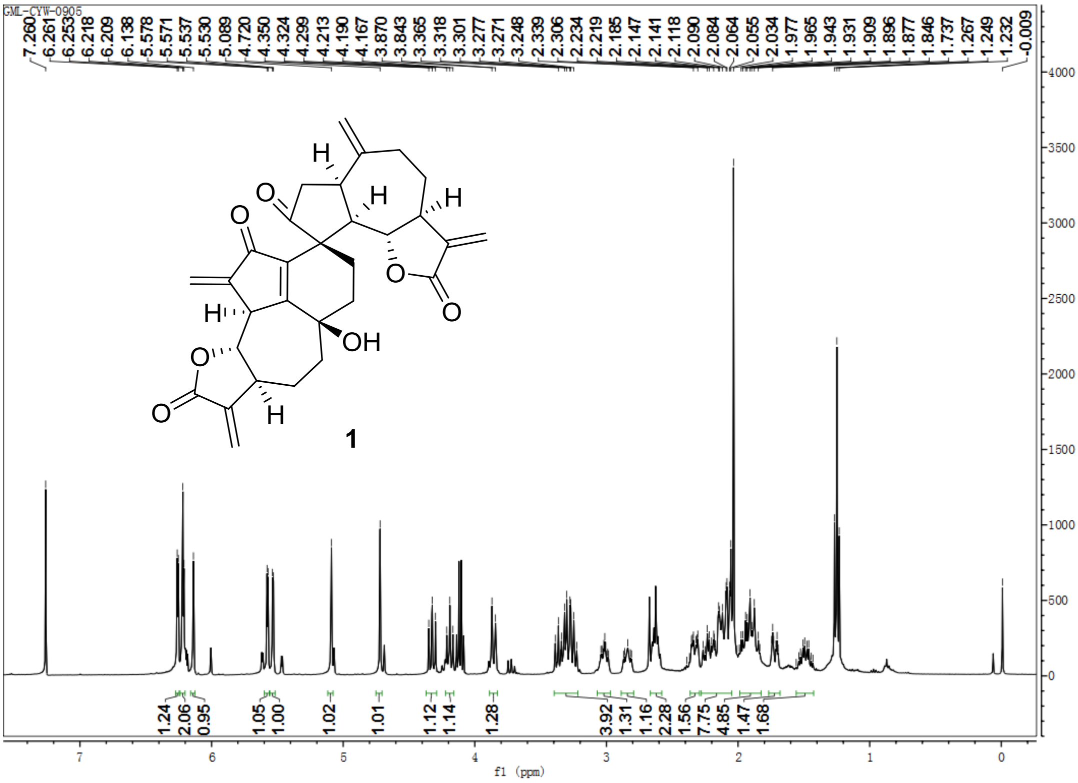

Supplemental Fig. 2 ^{1}H NMR spectra of (–)-gochnatiolide B

Supplemental Fig. 3

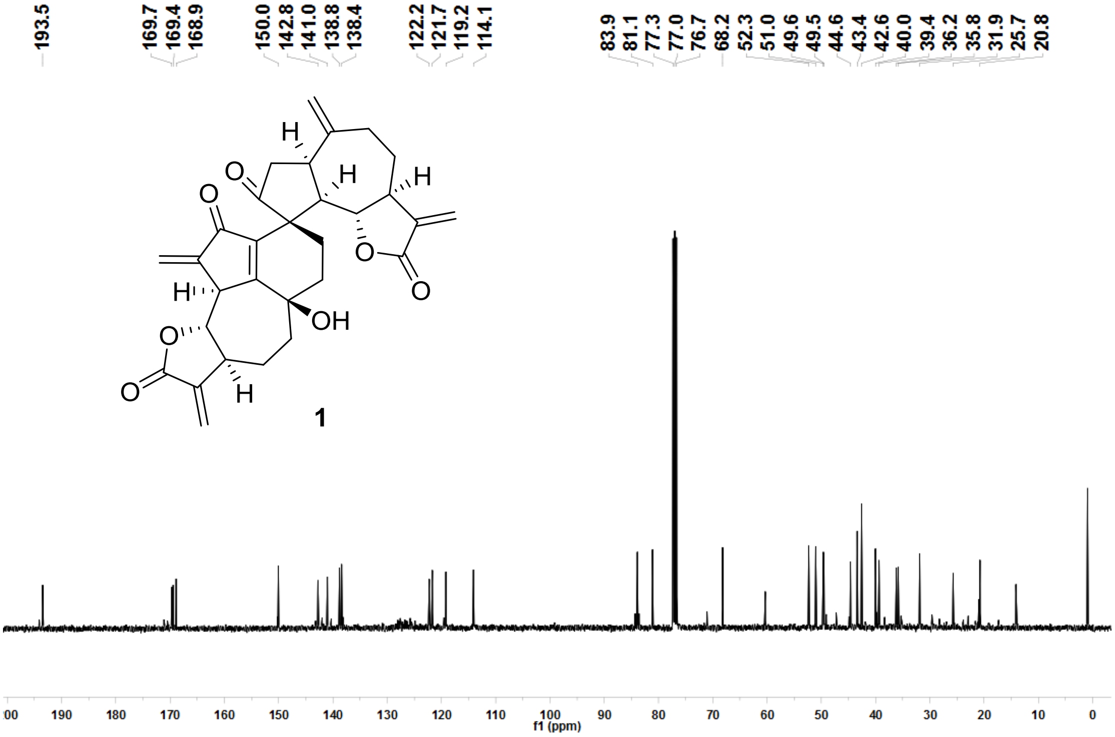

Supplemental Fig. 3 ^{13}C NMR spectra of (–)-gochnatiolide B

Supplemental Fig. 4

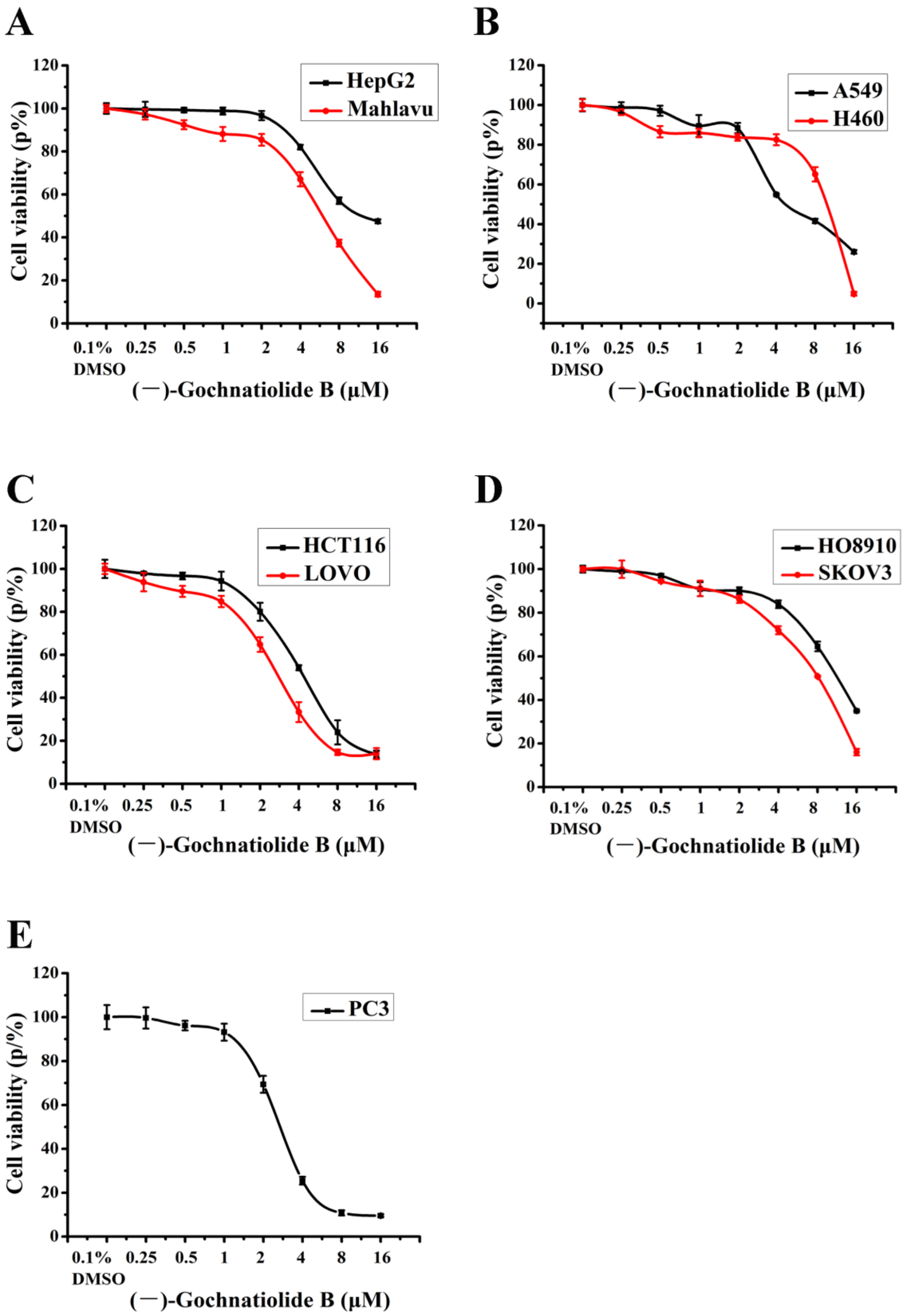

Supplemental Fig. 4 Growth inhibitory effects of (–)-gochnatiolide B on Liver (HepG2 and Mahlavu; **A**), lung cancer cells (A549 and H460; **B**), colon (HCT116 and LOVO; **C**), ovarian (LOVO and HCT116; **D**) and prostate (PC3; **E**) cancer cells. Cells were exposed to increasing concentrations of (–)-gochnatiolide B as indicated for 48 h respectively and cell viability was measured by MTT assay.

Supplemental Table 1

The IC_{50}s of (–)-gochnatiolide B on different cancer cell lines

Panel Name	Cell Line	IC_{50} (μM)
Liver	HepG2	12.4
	Mahlavu	6.4
Lung	H460	9.9
	A549	6.4
Ovarian	HO8910	11.2
	SKOV3	7.5
Bladder	EJ	2.7
	T24	3.7
	5637	3.0
	J82	4.0
	RT4	4.1
Colon	LOVO	2.8
	HCT116	4.2
Prostate	PC3	3.0

The IC_{50} values were calculated by SPASS 18.0
